# Characterizing organic particle impacts on inert metal surfaces: Foundations for capturing organic molecules during hypervelocity transits of Enceladus plumes

**DOI:** 10.1111/maps.13448

**Published:** 2020-02-25

**Authors:** J. S. New, R. A. Mathies, M. C. Price, M. J. Cole, M. Golozar, V. Spathis, M. J. Burchell, A. L. Butterworth

**Affiliations:** ^1^ Space Sciences Laboratory University of California Berkeley 7 Gauss Way Berkeley California 94720 USA; ^2^ School of Physical Sciences University of Kent Canterbury Kent CT2 7NH UK; ^3^ Department of Chemistry University of California Berkeley California 94720 USA

## Abstract

The presence and accessibility of a sub‐ice‐surface saline ocean at Enceladus, together with geothermal activity and a rocky core, make it a compelling location to conduct further, in‐depth, astrobiological investigations to probe for organic molecules indicative of extraterrestrial life. Cryovolcanic plumes in the south polar region of Enceladus enable the use of remote in situ sampling and analysis techniques. However, efficient plume sampling and the transportation of captured organic materials to an organic analyzer present unique challenges for an Enceladus mission. A systematic study, accelerating organic ice‐particle simulants into soft inert metal targets at velocities ranging 0.5–3.0 km s^−1^, was carried out using a light gas gun to explore the efficacy of a plume capture instrument. Capture efficiency varied for different metal targets as a function of impact velocity and particle size. Importantly, organic chemical compounds remained chemically intact in particles captured at speeds up to ~2 km s^−1^. Calibration plots relating the velocity, crater, and particle diameter were established to facilitate future ice‐particle impact experiments where the size of individual ice particles is unknown.

## Introduction

Studying the organic history, habitability, and potential for, or the presence of, extinct or extant life on any solar system body is an exciting, but challenging, quest. Instruments that can capture materials from plumes, clouds, comae, or ejecta and perform sensitive analyses for organic molecules advantageously avoid the technical and planetary protection complications of a surface lander. In this regard, the ice plumes emanating from Enceladus have recently attracted a great deal of attention. Here, we experimentally explore the feasibility of hypervelocity organic particle capture using a series of capture surfaces (CS). This work forms part of a project developing the Enceladus Organic Analyzer (EOA) instrument for probing biosignatures in icy plumes (Mathies et al. 2017).

The Cassini mission revealed a number of distinct, narrow geysers (Waite et al. 2006; Porco et al. 2014) venting from four prominent, and warm, fractures in Enceladus’ south polar region that make Enceladus a compelling target for noncontact analysis. These geysers form plumes that extend thousands of kilometers into space and are responsible for Saturn's E‐ring (Spahn et al. 2006). Cassini data (e.g., Postberg et al. 2011) and ground‐based telescope analysis of Saturn's E‐ring (Schneider et al. 2009) suggest that the largest particles near the surface of Enceladus (2–6 μm diameter at an altitude of 50 km) are frozen droplets of salty liquid water (0.5–2.0% NaCl by mass) and that vapor in the plume includes trace amounts of ammonia and light organic compounds (Waite et al. 2009). Furthermore, recent studies report the observations of emitted ice grains containing concentrated and complex macromolecular organic material with molecular masses above 200 atomic mass units (Postberg et al. 2018; Khawaja et al. 2019). Various sources of evidence indicate that the plume originates from a sub‐ice‐surface liquid water ocean, with salinity similar to oceans on Earth, that is in contact with a rocky core (Postberg et al. 2009). Predictive models (Zolotov 2007) and analysis of E‐ring particles (Hsu et al. 2015) indicate hydrothermal sources at the ocean‐core boundary, similar to the hydrothermal vents at Lost City (Kelley et al. 2005) on the Earth. The presence and accessibility of the salty liquid ocean, together with geothermal activity on Enceladus, make it a most promising place to conduct further, in‐depth, astrobiological investigations using remote in situ analysis techniques to probe for organic molecules indicative of extraterrestrial life.

A variety of in situ instruments have been developed for probing organic molecules and biosignatures in planetary science, particularly probing the environment of Mars. The Sample Analysis at Mars instrument uses gas chromatography‐mass spectrometry (GC‐MS) to measure light isotopes (H, O, C, N), volatiles, and to search for organic compounds directly from the atmosphere and any thermally released from solid samples (Mahaffy et al. 2012). The Mars Organic Molecule Analyzer (MOMA) onboard the ExoMars 2020 Rosalind Franklin rover will use GC‐MS and laser desorption mass spectrometry for organic analysis (Goesmann et al. 2017). The Scanning Habitable Environments with Raman and Luminescence for Organics and Chemicals (SHERLOC) instrument onboard the NASA Mars 2020 rover will use a deep ultraviolet Raman and fluorescence spectrometer that can characterize organic materials and will attempt to assess habitability and search for potential biosignatures (Beegle and Bhartia 2016).

The EOA instrument (homepage located at http://eoa.ssl.berkeley.edu as of October 2019) currently in development at UC Berkeley Space Sciences Laboratory is focused on the engineering of microfluidic chemical analysis flight systems with very high organic sensitivity and specificity based on the technology first developed, optimized, and field tested in the Mars Organic Analyzer (Skelley et al. 2005, 2007; Chiesl et al. 2009). For in situ studies of Enceladus, this technology has the advantages of small mass and size, autonomous operation, including fluidic manipulation, and high sensitivity for a variety of organic molecules that could be indicative of biosignatures. However, the requirement for efficient plume sampling and transport of the captured organic materials to the organic analyzer present unique challenges for an Enceladus mission.

Depending on the orbital navigation (e.g., an Enceladus or Saturn orbit), plume encounter speeds could range from a few hundred m s^−1^ to several km s^−1^, and fall into the “hypervelocity” regime. During hypervelocity impacts, both projectile and target undergo significant disruption and/or modification (e.g., Avdellidou et al. 2015 and Wickham‐Eade et al. 2018). This is due to shock pressures of the order of GPa (depending on the impact speed) and high temperatures (that can reach thousands of K, albeit for a brief amount of time) that are indicative of such impacts (Melosh 1989). Organic compounds are sensitive to both excessive shock and prolonged heating, both of which can alter bonds (Goldman et al. 2010), and thus, the physical conditions that occur during an impact must be carefully considered when designing an organic material capture system.

There has previously been an extensive literature on survival after hypervelocity impacts on metal targets of non‐organic materials, such as metal and mineralic projectiles. For example, Bernard and Hörz (1995) show how residue from glass impactors survives in impacts on aluminum at speeds up to 7 km s^−1^. For some minerals, Burchell et al. (2008) showed that residue can not only survive but also it can still give a recognizable Raman signature, implying the internal structure has been preserved. Subsequent work showed that the Raman signal can be altered slightly as a result of the impact (Foster et al. 2013; Harriss and Burchell 2016). It is possible that the minerals in such impacts recrystallize after melting, but this was shown not to be the case (Wozniakiewicz et al. 2012) implying that at least some of the original projectile material survives relatively unaltered.

A number of studies demonstrate the capture and survival of organic compounds during hypervelocity impacts. For example, Parnell et al. (2010) successfully demonstrated the survival of organic biomarkers in craters and ejecta after hypervelocity impacts with velocities ranging between 2 and 6 km s^−1^. Price et al. (2012) explored the creation, destruction, and modification of organic species during hypervelocity impacts of polystyrene particles and found that Raman signatures of residual polystyrene were observed in craters after ~1.5 and ~6.1 km s^−1^ impacts, but at higher speeds, the majority of residue was elemental carbon. Burchell et al. (2014) observed successful transfer of organic compounds from projectile to target during hypervelocity impacts at velocities of ~2 and ~4 km s^−1^. Burchell and Harriss (2019) examined the survival of organic particles after impacts at a velocity of 5–6 km s^−1^ with the goal of differentiating between aromatic and aliphatic chemical structures in the captured projectile. NASA's Stardust mission (Brownlee et al. 1997, 2006) exposed a collector made of silica aerogel and aluminum foil during passage through the tail of Comet Wild‐2 at an encounter velocity of 6.1 km s^−1^. Detection of organic material (Glavin et al. 2008; Clemett et al. 2010) was observed inside several of the impact tracks on the aerogel collector. Although first thought to be potentially of terrestrial origin (Glavin et al. 2008), cometary glycine was found on the comet‐exposed aluminum foils (Elsila et al. 2009), which is a more representative material of those expected on an Enceladus capture mission. Furthermore, polycyclic aromatic hydrocarbons, most likely of cometary origin, that were unambiguously associated with impact residues were also identified on the foils (Leitner et al. 2008). A recent study by Sekine et al. (2018) predicts that particle capture at 3 km s^−1^ velocity with shock pressures of 8 GPa and temperatures of 300 °C is feasible for Enceladus. All of these observations would lead us to suggest that a speed of ~6 km s^−1^ is the very highest limit for detectable survival of organic compounds onto a metallic (aluminum) collector.

These extensive results demonstrate that organic molecules do survive hypervelocity impacts, but the extent of (unmodified) particle capture is unquantified. Although the amount of surviving impactor residue decreases as a function of impact velocity, at speeds of 6 km s^−1^ the quantity of surviving residue becomes extremely difficult to detect (Kearsley et al. 2010), and the degree of residue alteration is also unknown. Furthermore, the relationship between organic survival (and/or alteration), capture efficiency, impact velocity, and capture medium is not well defined, indicating the critical need for a detailed experimental study of these parameters.

To address this problem, we performed a systematic study where organic ice‐particle simulants, with diameters of 4, 6, and 10 μm, were accelerated into soft inert metals at velocities of ~0.5, 1.0, 2.0, and 3.0 km s^−1^ with the objective of answering four fundamental questions. (1) How does the nature of impacts change with respect to velocity and target material? (2) What is the optimal material for effective particle capture during the specified impact velocities? (3) Do organic ice‐particle simulants remain chemically intact during the impacts? (4) What is the particle–crater diameter relationship at the specified impact velocities for the target materials?

## Experiments and Methodology

**Figure 1:**
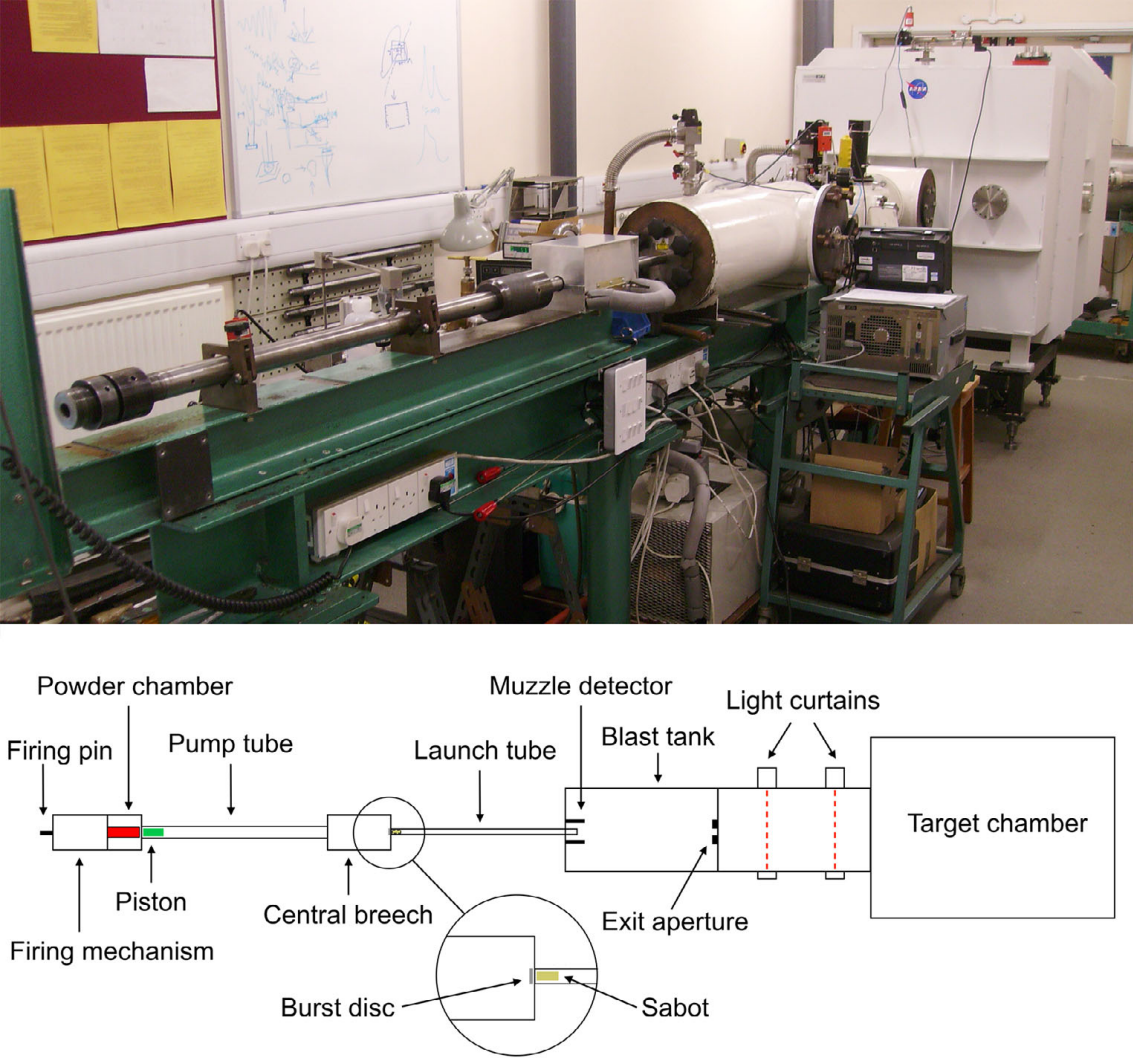
Photograph (top) and schematic (bottom) of the light gas gun (LGG) at the University of Kent. A shotgun cartridge detonates, which drives a piston that compresses a light gas in the pump tube. A thin aluminum disc ruptures at a specific pressure (~2 kbar) allowing the compressed gas to accelerate the projectile (housed in a sabot) through an evacuated launch tube. Light curtains and impact sensors are used to measure the velocity. The target is positioned in either the blast tank or target chamber—both of which are evacuated.

A series of high‐velocity and hypervelocity impact experiments were carried out using the light gas gun (LGG) at the University of Kent (described by Burchell et al. 1999; Hibbert et al. 2017), as seen and illustrated in Fig. 1. This particular LGG facility was selected due to its ability to accelerate a wide range of particles, both size and composition, up to velocities of ~7.5 km s^−1^ and offer flexibility in target configuration and temperature. Most critically, however, the LGG at Kent offers the unique capability of accelerating ice projectiles, which will be necessary for the second stage of this research and the EOA development.

Targets were designed to study the CS materials of interest. These materials were chosen to provide a compliant impact surface that can effectively capture particles and reduce shock and thermal destruction of the organic compounds in the projectiles. Additionally, the materials had to permit fabrication in a variety of configurations and thicknesses, suitable for space‐flight instrument deployment. Finally, they had to be readily cleaned to very low levels of organic contamination and easily washed to extract captured particle residue for analysis. Thus, soft, relatively inert metal foils were chosen for testing, including aluminum, copper, gold, indium, and silver (supplier and catalog details in Table S1 the supporting information). Five foils, one of each material, were configured in a 3/2 grid measuring ~2 × 2 cm and were adhered to a carbon pad attached to a 6 mm thick aluminum disc. This design acted as a control allowing the projectiles to impact each target metal under nominally similar impact conditions for direct comparison.

Polymethylmethacrylate (PMMA) particles were selected as the ice‐particle simulants for the experiments as they are solid at room temperature and have an accessible melting point (160 °C) and density (1.18 g cm^−1^) similar to ice. The majority of the Enceladus plume mass, at an altitude of 50 km, resides in particles with a diameter of 4–6 μm (Hedman et al. 2009) and monodisperse PMMA particles of this size are readily available. Particles with diameters of 4 and 6 μm were selected to simulate plume particles, while additional 10 μm particles were included to extend the data set. Monodisperse PMMA particles were selected in order to develop accurate particle–crater diameter calibration plots for the specified impact velocities on the different target materials. Finally, the mechanical profiles of PMMA polymers are well defined enabling the development of hypervelocity impact hydrocode models.

During each experiment, thousands of projectiles, either 4, 6, or 10 μm diameter, were loaded into a sabot and fired onto the metal targets. The sabot was discarded in flight, leaving the projectiles to proceed to the target. The velocities of the projectiles (~ 0.5, 1.0, 2.0, and 3.0 km s^−1^) were measured from their time of flight between a laser curtain at the end of the launch tube and a piezoelectric impact sensor attached to the target. As a result, velocity measurements were accurate to ±0.01 km s^−1^. The blast tank was maintained at a vacuum of 0.5 mbar throughout the experiments to prevent slowing of the projectiles due to air resistance and the targets were placed into the blast tank of the LGG at normal incidence to the projectiles’ flight path.

A minimum of 10 impact craters on each of the target materials were analyzed after every experiment. A field emission scanning electron microscope (FEG‐SEM, Hitachi S‐4700) was used to obtain high resolution images of impact craters on the targets. Energy dispersive X‐ray (EDX) microanalysis, using a Bruker Quantax FlatQUAD, was used to identify and detect the abundance of atomic carbon as a tracer for PMMA (C_5_O_2_H_8_), providing a means of assessing particle capture. The image processing software ImageJ (Abràmoff et al. 2004) was used to record the average diameter and area of the craters formed by the different sized particles at given velocities. These data were plotted in order to calculate the particle–crater diameter calibrations. Micro‐Raman spectroscopy, using a LabRam‐HR from Horiba incorporating 632 nm excitation laser and 1000× magnification, was used to analyze the organic compounds within the captured material and determine organic survival. The Raman signature of PMMA has a number of well‐defined peaks that act as a “fingerprint” that were directly compared with the spectra of captured material to confirm the presence of intact/unmodified PMMA.

## Results

The capture efficiency, organic survival, and particle–crater diameter calibration results are presented in separate sections and the nature of the impacts are described for each particle size and velocity. Tables of data are provided in the supporting information.

### Capture Efficiency

The relative capture efficiency of each CS material was quantitatively measured by calculating the mean residue coverage (MRC), described as the ratio between crater area and residue area, per crater across a sample of 25+ craters. This method was selected due to the challenges associated with measuring the volume of residue deposited on the targets, as accurately determining the thickness of the residue (~50 nm–5 μm) is extremely challenging and destructive. Capture efficiency was primarily affected by the impact velocity and CS material and declined with increasing impact velocities. Capture efficiency varied between the different particle sizes and CS, but maintained a similar trend.

#### 0.5 km s^−1^ Velocity Impacts

**Figure 2:**
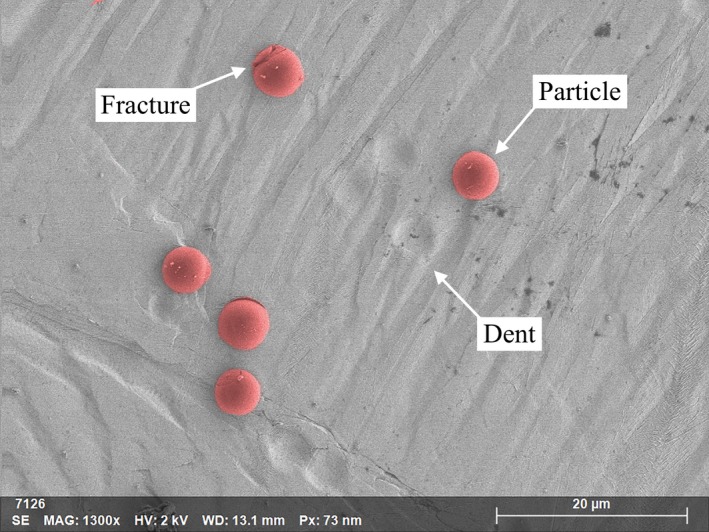
SEM image of the indium capture surface after impacts with 4 μm diameter PMMA particles (false color red) at a velocity of 0.54 ± 0.01 km s^−1^. Particles and dents are observed on the foil, with most particles exhibiting no detectable deformation and one with a minor fracture in the top of the image.

During the 0.5 km s^−1^ velocity impacts, the particles either stuck to the targets or rebounded, in some cases leaving dents, illustrated in Fig. 2. Typically, very little particle deformation was observed and most of the captured particles maintained their original spherical shape and size, with a small number of particles exhibiting minor fractures (Fig. 2).

The sticking coefficient, defined simply as the ratio of stuck‐particles‐to‐dents, was used to calculate the capture efficiency, where the MRC was 100% or 0% for captured whole particles and dents, respectively. During the very low (0.5 km s^−1^) velocity impacts, it was only possible to directly calculate the sticking coefficient for the indium foil as the harder CS materials did not deform. Due to the grid formation and close proximity of the foils on the target, a uniform impact distribution of particles was assumed, permitting calculations for the sticking coefficient on the remaining CS materials.

The 4 μm diameter particles were captured with the highest efficiency, relative to the other particle sizes, across all of the CS materials at this speed (see Fig. 6). The highest performing material was indium (66% MRC) and the lowest was aluminum (21% MRC), with an average capture efficiency of 43.4% across all the CS materials. The 6 μm diameter particles were captured with an average MRC of 4% across the CS materials and the 10 μm diameter particles had a similarly low capture efficiency with MRC ≤2% across the CS materials. This represents a significant decline in capture compared to the 4 μm particles.

#### 1.0 km s^−1^ Velocity Impacts

**Figure 3:**
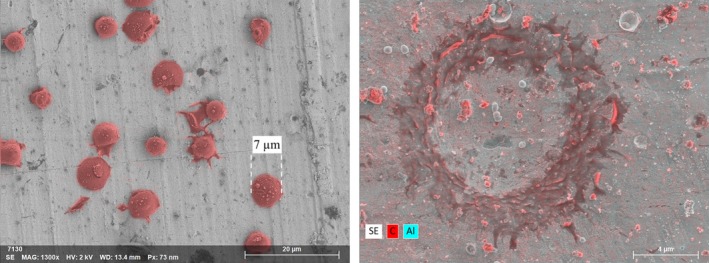
Left) SEM image of 4 μm (pre‐impact) diameter PMMA particles (false color red) stuck to the silver capture surface after a 0.85 ±  0.01  km s^−1^ velocity impact. A 7 μm scale bar has been positioned over one of the particles to highlight the resulting size deformation. Right) SEM‐EDX image of the silver capture surface foil after a 0.97 ± 0.01 km s^−1^ velocity impact with a 10 μm diameter particle that rebounded off the foil and deposited a ring of carbon‐rich residue (red) around the dent.

During the 1.0 km s^−1^ velocity impacts, the capture efficiency of the CS materials improved for all sizes of particles. The nature of the impacts was similar to those observed at 0.5 km s^−1^ velocity, where particles stuck to, or rebounded off, the target. Figure 3 (left) reveals that, although whole particles were captured on the target, many of the particles exhibited deformation and shape modification. Additionally, rings of carbon‐rich residue, presumed to be PMMA, were observed around a large number of dents, demonstrated in Fig. 3 (right). Dents were observed on all of the CS materials and, therefore, direct sticking coefficients were calculated for each material.

The 4 μm diameter particles were captured with an average MRC of 69.2% across the CS materials. The highest performing material was silver (85% MRC), with gold (84%) and indium (82%) exhibiting similarly high capture efficiency. The 6 μm diameter particles were captured with improved efficiency during the 1.0 km s^−1^ velocity impacts. Indium had the highest MRC (79%) and the lowest was aluminum (3%), with an average capture efficiency of 30.8% across the CS materials. The capture efficiency of the 10 μm diameter particles improved compared to the 0.5 km s^−1^ velocity impacts, but was still relatively low, with an average MRC of 5.8% with only gold and indium exhibiting notable particle capture.

#### 2.0 km s^−1^ Velocity Impacts

**Figure 4:**
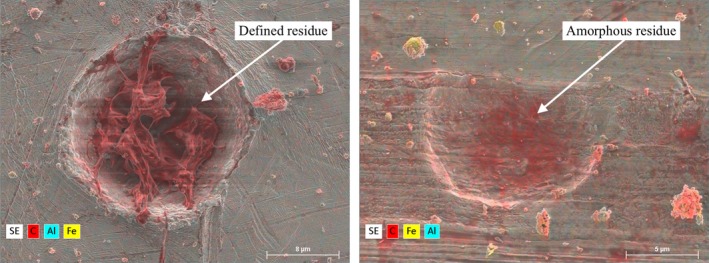
SEM‐EDX images of an impact crater and dent on indium (left) and copper (right) caused by impacts with 10 μm diameter PMMA particles at a velocity of 1.94 ± 0.01 km s^−1^. Well‐defined stringy carbon/PMMA residue (left) and amorphous residue (right) is highlighted in red.

Whole particles were no longer captured by the CS during the 2.0 km s^−1^ velocity impacts and target deformation increased to form impact craters. Well‐defined “stringy” residue—indicative of projectile melting—was observed in certain craters, illustrated in Fig. 4 (left), while others only contained amorphous residue concentrates, Fig. 4 (right). A number of craters exhibited no detectable residue capture. Both types of residue were deposited within the craters, and in some cases, extruded beyond the crater's rim.

The 4 μm diameter particles were captured with an average MRC of 12.4% across the CS materials. The highest performing material was silver (20.4% MRC) and the lowest was copper (3.7% MRC). The 6 μm diameter particles were captured with an average MRC of 12.9% across the CS materials. Indium had the highest capture efficiency (15% MRC) and aluminum had the lowest (7.5% MRC). The capture efficiency was significantly higher for the 10 μm diameter particle impacts, with an average MRC of 25.5%. Similarly, indium had the highest capture efficiency (35.3% MRC) and aluminum had the lowest (14.7% MRC).

#### 3.0 km s^−1^ Velocity Impacts

**Figure 5:**
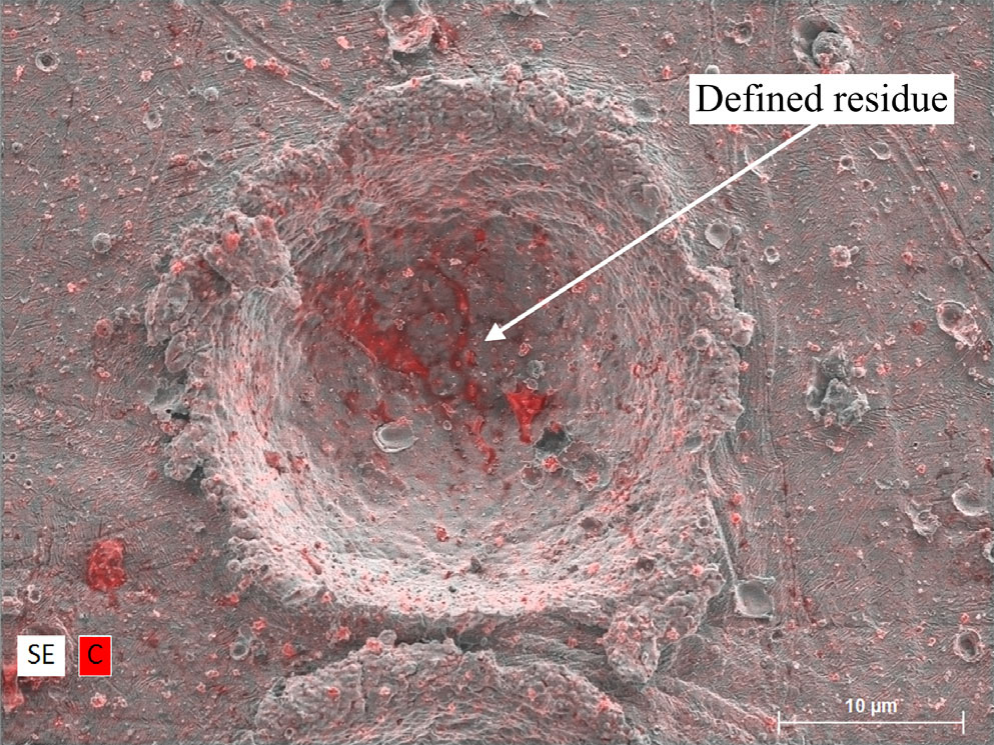
SEM‐EDX image of a crater from a 10 μm diameter PMMA particle on the indium capture surface after an impact at a velocity of 2.92 ± 0.01 km s^−1^. Well‐defined stringy carbon/PMMA residue is highlighted in red.

During the 3.0 km s^−1^ velocity impacts, the capture efficiency across the CS materials significantly decreased for all sizes of particles. The nature of impacts was similar to those observed at 2.0 km s^−1^ velocity, where particles were highly disrupted and deposited well‐defined stringy residue (Fig. 5) and amorphous concentrates on the CS, but in much lower quantities.

The 4 μm diameter particles were captured with an average MRC of 4.7% across the CS materials. The highest performing material was indium (7.5% MRC) and the lowest was gold (2.0% MRC). The 6 and 10 μm diameter particles had similarly low capture efficiency, with an average MRC of 0.4% and 0.7%, respectively. Indium had the highest capture efficiency and was the only CS material for both 6 and 10 μm diameter particles to have MRC >1% at 1.3% and 2.2%, respectively.

#### Summary of Velocities

**Figure 6:**
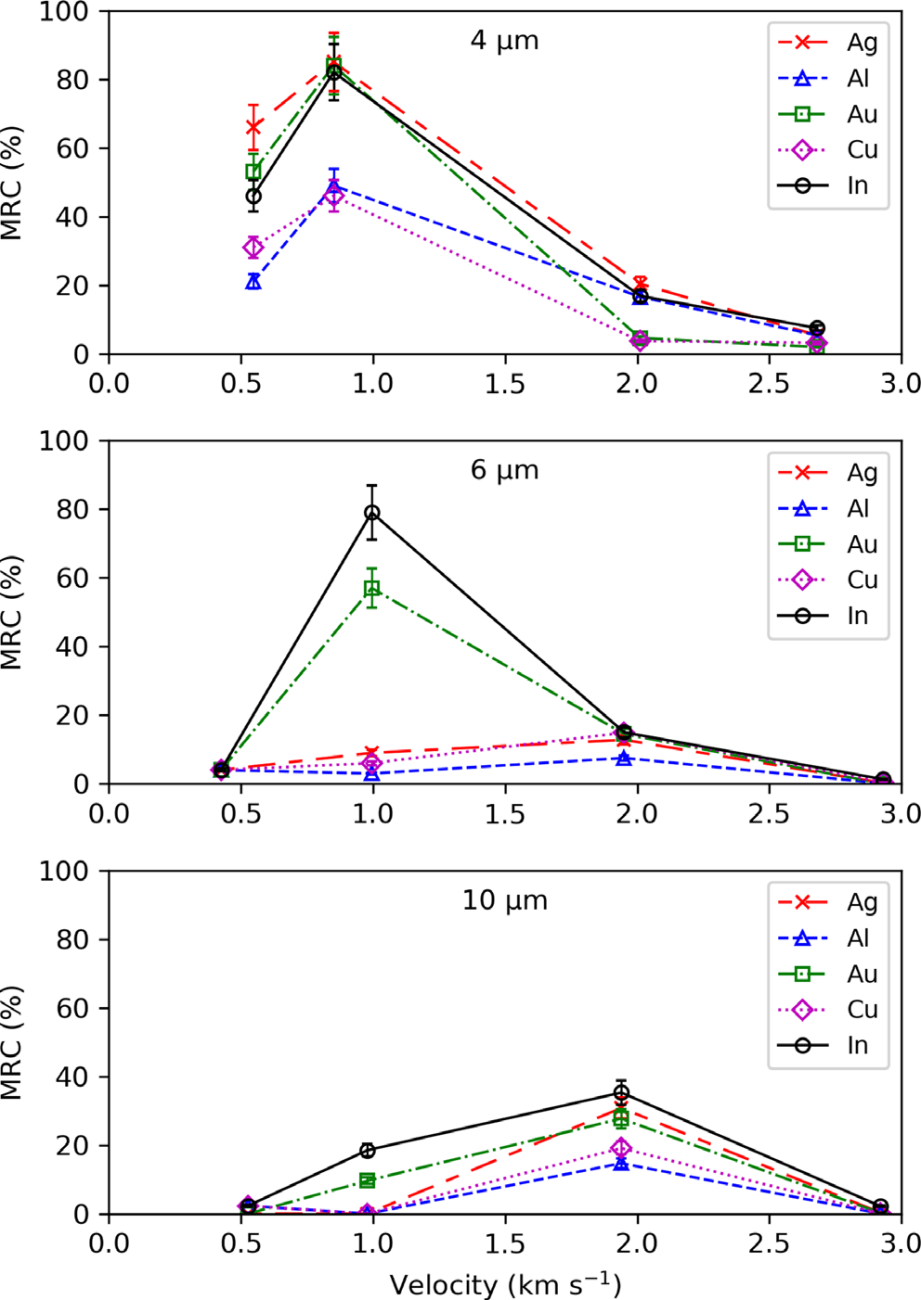
Mean residue coverage (MRC) plotted against impact velocity for Ag (red), Al (blue), Au (green), Cu (magenta), and In (black) for each particle diameter (4, 6, and 10 μm). Error bars (±10%) represent the uncertainty in the residue area and sticking coefficient due to manual measurements using ImageJ.

To summarize the capture efficiency data, the MRC was plotted against impact velocity for all the CS materials for each particle diameter (Fig. 6). The plots show that gold, indium, and silver represented the best capture mediums across the 0.5–3.0 km s^−1^ velocity range, with peak capture occurring at ~1.0 km s^−1^ for small particles and ~2.0 km s^−1^ for larger particles. A table of data is available in the supporting information.

### Organic Survival

**Figure 7:**
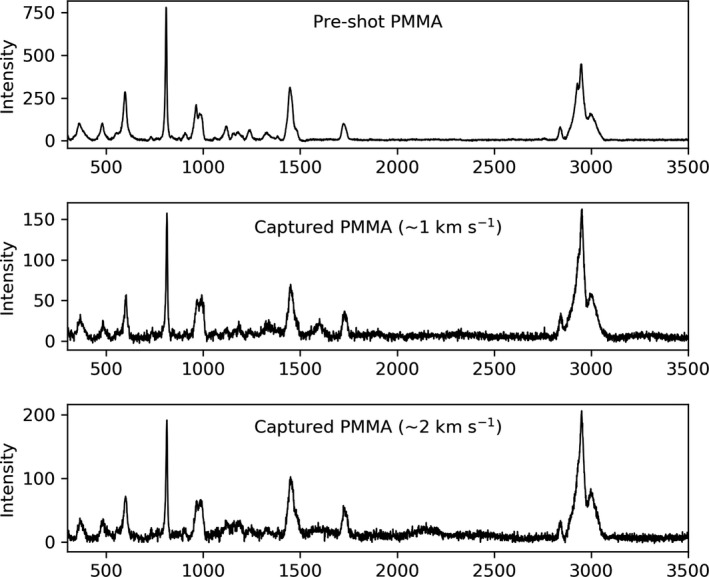
Raman spectra of pre‐shot PMMA particles (top), a particle captured on the indium capture surface at 0.99 ± 0.01 km s^−1^ velocity (middle), and residue captured in a crater on the indium target at 1.94 ± 0.01 km s^−1^ velocity (bottom). The spectra indicate that captured PMMA remains chemically intact up to ~2 km s^−1^ velocity.

The vibrational spectra of pre‐shot PMMA particles were analyzed using a Raman spectrometer to facilitate direct comparison between captured particles and residues (Fig. 7). Peaks in the pre‐shot PMMA were observed at 487 cm^−1^ (C‐C in plane bending), 600 cm^−1^ (C‐O in plane bending), 810 cm^−1^ (C=O in plane bending), 970 cm^−1^ (C‐C stretching), 990 cm^−1^ (C‐C stretching), 1453 cm^−1^ (CH_3_ deformation), and 1730 cm^−1^ (C=O stretching). These correlate with literature for observed and theoretical Raman wave numbers for PMMA (Haris et al. 2010).

Raman spectral analysis for the 1.0 and 2.0 km s^−1^ velocity impacts revealed that the Raman spectra were unchanged between the pre‐shot PMMA and the particles and residues captured by the CS—confirming PMMA remains chemically intact and unmodified under these impact conditions. Figure 7 provides a direct comparison among (1) pre‐shot PMMA particles, (2) a particle captured on the indium target at 0.99 ± 0.01 km s^−1^ velocity, and (3) residue captured in a crater on the indium target at 1.94 ± 0.01 km s^−1^ velocity. The low capture efficiency at ~3.0 km s^−1^ meant Raman microscopy was inconclusive for residue identification within the craters.

### Particle–Crater Diameter Calibration

**Figure 8:**
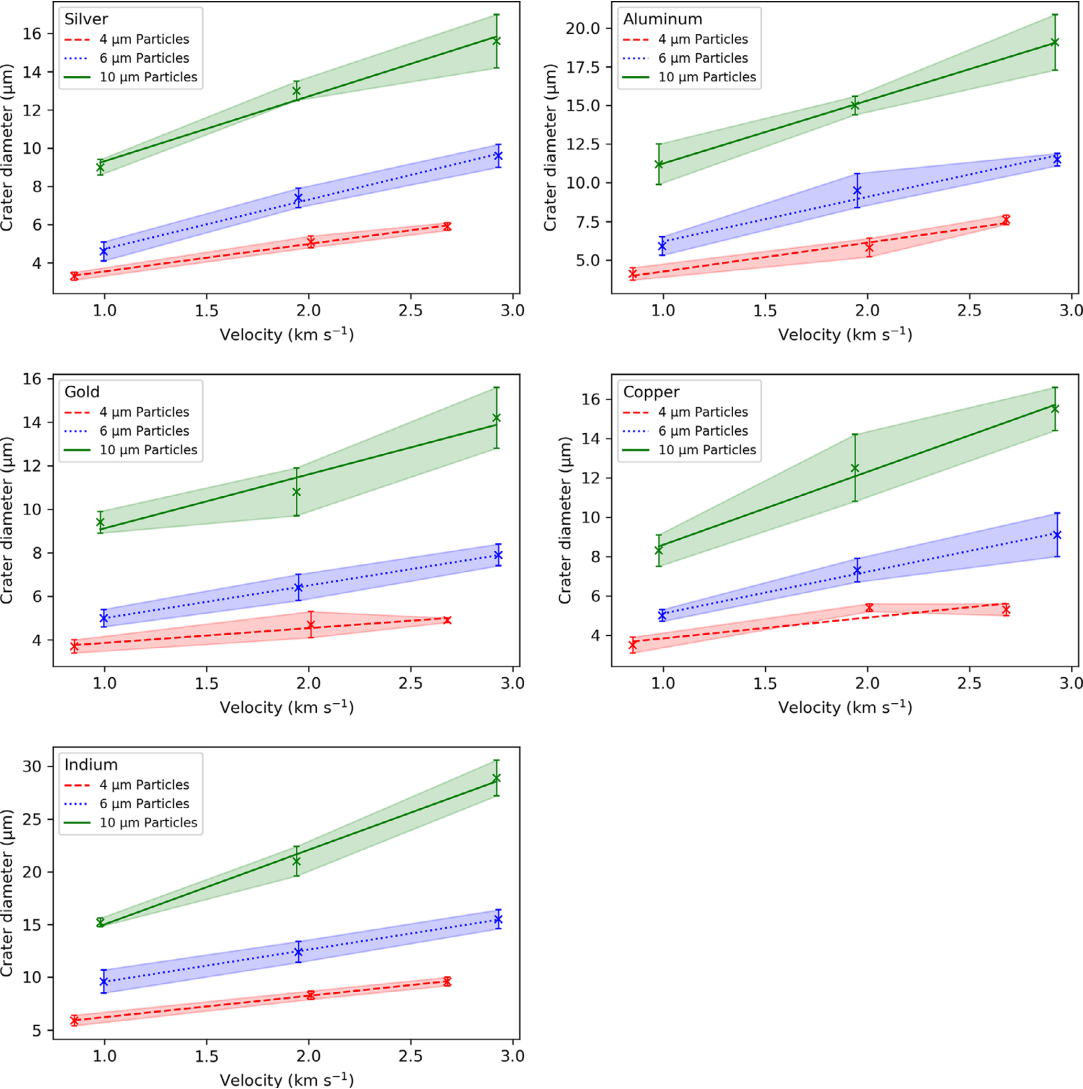
Velocity–crater diameter calibration plots for ice simulants (PMMA) impacting the CS materials at velocities ranging 1.0–3.0 km s^−1^. Trend lines are plotted for the different particle diameters and shaded regions represent the standard deviation (1σ, *n* = 10) of the mean crater diameters.

The relative size of the craters, with respect to particle diameter and impact velocity, was measured by averaging the major and minor diameters from the best‐fit‐ellipse for a sample of 10 craters on each CS material. This method provided a means of calculating the size of circular and irregularly shaped impact craters. The mean crater diameter was plotted against impact velocity for each of the particle diameters and CS materials (Fig. 8). The error bars and shaded regions represent the SD (1σ, *n* = 10) of the crater diameters. A linear correlation between the crater diameter and impact velocity for each particle size and CS material was observed and is represented on the plots with a trend line.

**Figure 9:**
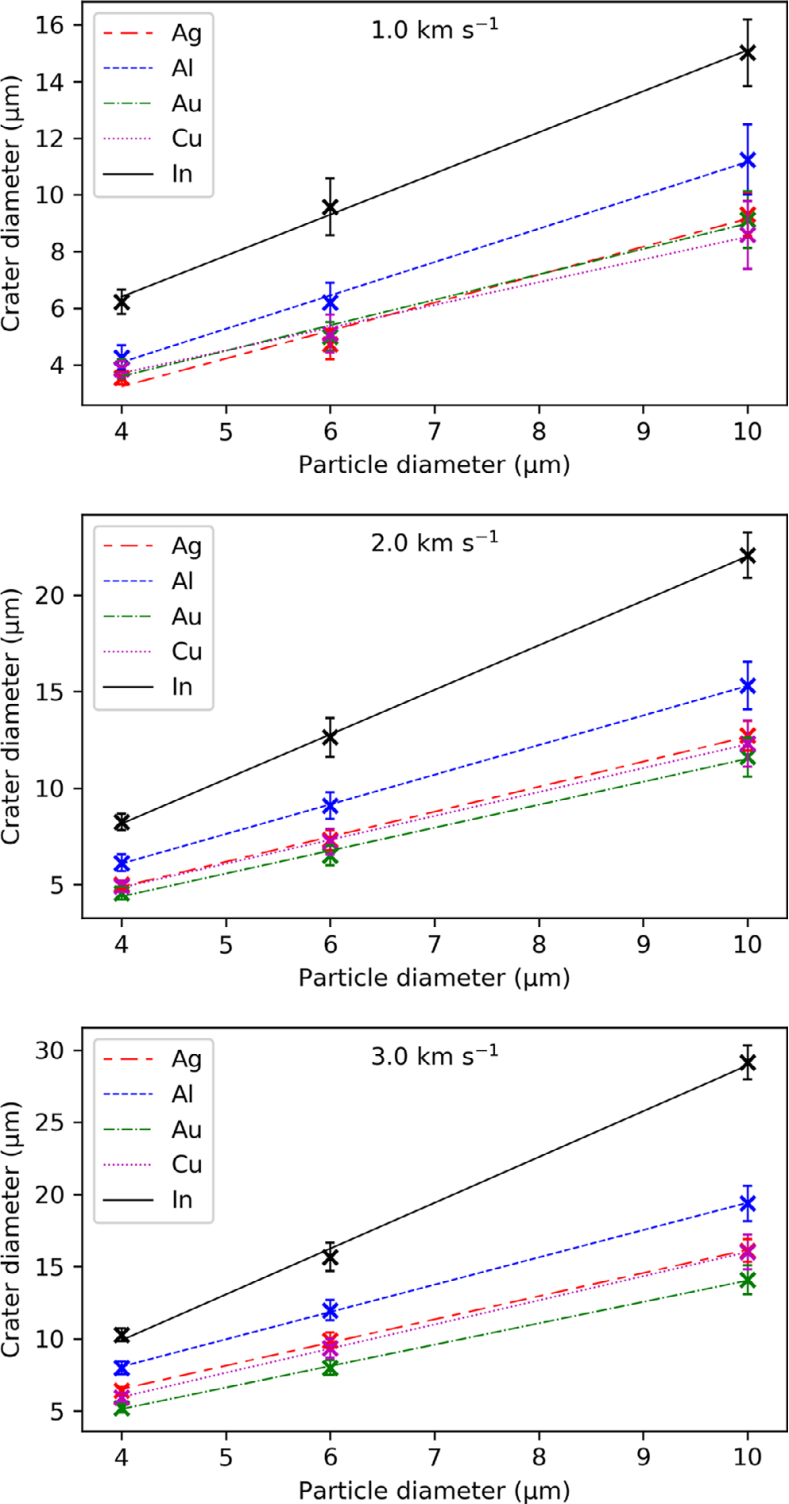
Particle–crater diameter calibration plots for ice simulants (PMMA) impacting Ag, Al, Au, Cu, and In targets with 1.0, 2.0, and 3.0 km s^−1^ velocity. Error bars represent the uncertainty derived from the mean standard deviation (1σ) on the velocity–crater diameter calibration plots across the 1.0–3.0 km s^−1^ velocity range for each particle size (4, 6, and 10 μm).

The trend lines from the velocity–crater diameter plots can be interpolated at any given velocity to ascertain the particle–crater diameter calibration for each CS material. Values for the *y*‐intercept (a) and gradient (b) for each CS material and particle size are included in supporting information and indicate that the crater diameter increases with speed and at an increasing rate with respect to particle diameter. Particle–crater diameter calibration plots for the velocities studied are provided in Fig. 9. The error bars represent the uncertainty carried forward from the velocity–crater diameter plots and are the mean standard deviation (1σ) across the 1.0–3.0 km s^−1^ velocity range for each particle diameter. A linear correlation between the particle and crater diameter is observed. Extrapolation beyond the 1.0–3.0 km s^−1^ velocity range is not advised as craters were not reliably observed below 1.0 km s^−1^ and above 3.0 km s^−1^ the particle–crater diameter relationship may become nonlinear.

The particle–crater diameter calibration plots will facilitate ice‐particle impact experiments by providing a means of calculating the diameter of an impactor from the crater it creates on impact with a specific target material at a given velocity.

## Discussion

Our overall goal is to explore the feasibility of flying through the ice plumes at Enceladus and gathering sufficient ice particles that we can perform a sensitive analysis for unmodified organic biomarkers using, for example, the EOA (Mathies et al. 2017). It is thus important to understand the nature of particle impacts on different target materials, to understand how the impacts change with velocity and particle size, to identify the optimal material for particle capture, and to ascertain the organic survival of ice‐particle simulants postimpact. We begin here by studying a model ice particle—PMMA—because this polymer has mechanical properties and a phase transition that are similar to ice and it is available in a wide variety of well‐defined diameters. These PMMA impacts establish a particle–crater diameter calibration that will facilitate the interpretation of ice‐particle impact experiments in the next phase of this research. Therefore, it was important to select a material with a phase transition at a relevant temperature (160 °C for PMMA) as this not only affects the capture efficiency but also may contribute to the deposition of organic compounds entrained in ice particles.

We performed LGG experiments to study the impacts of PMMA particles into a selection of inert metal target CS at different velocities. Organic PMMA ice‐particle simulants with diameters of 4, 6, and 10 μm were chosen to imitate the size of particles in Enceladus’ plume at an altitude of 50 km. Impact velocities ranging from 0.5 to 3.0 km s^−1^ were selected. Velocities of 0.5 km s^−1^ are the practical lower limit of the experiments and approach the velocity of an Enceladus orbit. Velocities of 3.0 km s^−1^ represent a possible Saturn orbiter. Inert metal target foils of Ag, Al, Au, Cu, and In were identified as potentially compliant capture materials that meet the science and engineering requirements of the EOA capture system (Mathies et al. 2017). In particular, we desire a compliant material that more gradually slows the impacting particle and creates a crater to capture the residue. We also desire a material that can be easily cleaned to provide low background organic levels. Furthermore, the CS must be easily washed to release the captured materials for analysis. Both of these desirements are not easily achieved by low‐density porous capture materials such as aerogels (Burchell et al. 2006). Our results reveal that organic compounds do survive impacts in the velocity range studied and that capture efficiency is influenced by the CS, impact velocity, and particle size.

At the low velocity 0.5 km s^−1^ range, whole particles were captured during the impacts with capture efficiency depending on size. SEM‐EDX analysis revealed that the captured particles experienced little or no deformation. This suggests that the particles did not melt and fuse to the CS on impact, but rather sticking of the particle onto the cratered target was responsible for capture. This hypothesis is supported by the absence of detectable residue in the dents imprinted on the target by rebounded particles. Interestingly, significant capture was only observed for the 4 μm diameter particles, suggesting that the stiction force acting on the heavier 6 and 10 μm particles parallel to the vertically mounted CS was insufficient to keep them bound to the surface. Alternatively, the larger particles with higher kinetic energy rebound off the target with great enough force to overcome the stiction. Gold, indium, and silver had the highest capture efficiency of the CS materials for the 0.5 km s^−1^ velocity impacts. This result is reasonable as the softer materials would yield larger impact craters that would provide greater stiction due to increased contact area between the CS and the particles. This stiction could be due to van der Waals interactions between the PMMA and the metals, but it may also be due to partial melting of the PMMA surface; however, there was no evidence of sufficiently thick molten residue in the craters to confirm this hypothesis for the 0.5 km s^−1^ velocity impacts.

At higher 1.0 km s^−1^ velocity, the capture efficiency increased for all of the CS materials. This trend could be explained by the increased stiction force between the CS and the particles due to deeper surface penetration and enhanced surface contact area. An alternative, or perhaps additional, hypothesis is that these higher velocity impacts have sufficient energy to partially melt the particles causing them to fuse with the target and improve capture. It is therefore expected that CS materials with low thermal conductivity would provide a better capture medium due to increased melting as less thermal energy dissipates from the particles to the target. This hypothesis is supported by the results where indium, the CS with the lowest thermal conductivity (83.7 K), had the highest increase in capture efficiency relative to the other CS materials. Furthermore, SEM‐EDX analysis indicated that particles captured on the indium target underwent the highest thermal deformation. However, since impacts into softer indium produce deeper and larger craters we would expect an enhanced stiction process for indium as well. A similar relationship between the particle size and capture efficiency was observed for the 1.0 km s^−1^ velocity impacts, where the 4 μm diameter particles were captured with highest efficiency, followed by the 6 and 10 μm diameter particles.

**Figure 10:**
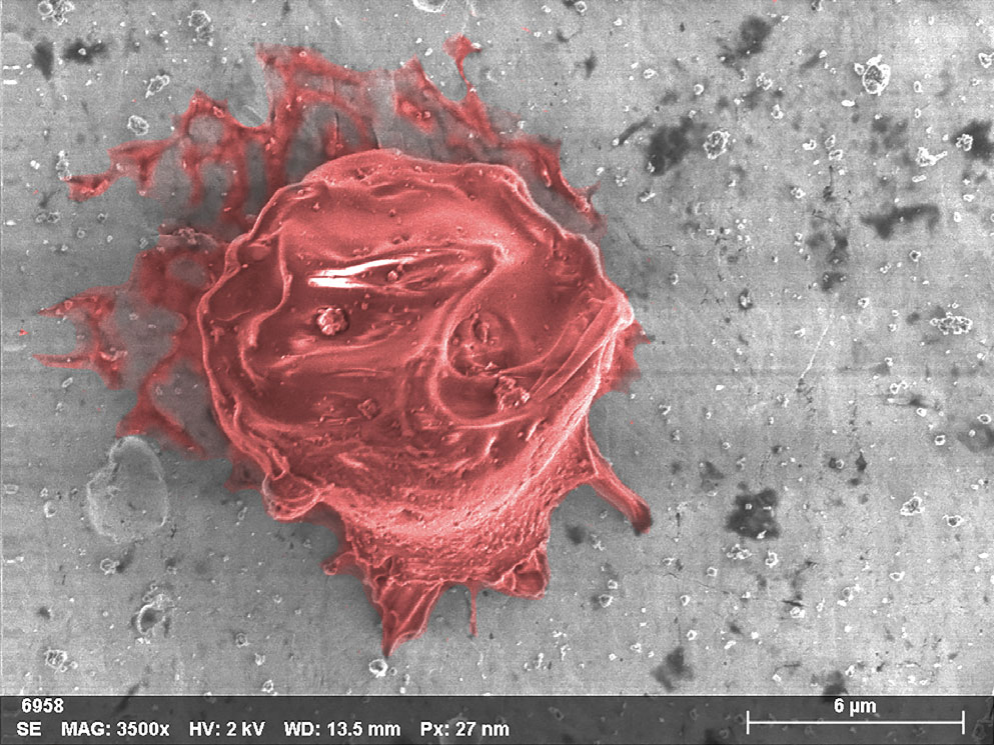
SEM image of a 10 μm diameter PMMA particle (false‐color red) captured by gold foil after a 0.97 ± 0.01 km s^−1^ velocity impact. Thermal deformation of the particle and extruded residue deposited on the foil surrounding the particle can be observed.

To explore the hypothesis that the particles are melting in the 1.0 km s^−1^ velocity impacts, we calculated that the energy required to melt a 10 μm diameter PMMA particle starting at room temperature was equal to ~1.2 × 10^−7^ J. It is generally accepted that during an impact, the energy is roughly distributed evenly between the target and the projectile (Gault and Heitowit 1963), where energy remaining in the projectile dissipates via heating and physical deformation. Individual PMMA particles with a 10 μm diameter and 1.0 km s^−1^ velocity have a kinetic energy of ~3.1 × 10^−7^ J. Assuming 50% of the kinetic energy is transferred to the target, ~1.6 × 10^−7^ J of the energy would remain in the projectile. This suggests that the energy required to heat the whole PMMA particle to melting point (160 °C) from room temperature is approximately 75% of the energy deposited in the particle. It is expected that the remaining (25%) energy dissipates through other means such as particle deformation. This result is supported by SEM analysis that reveals thermal alteration in the particles (Fig. 10) during the 1.0 km s^−1^ velocity impacts and signs of molten residue deposits around the perimeter of numerous craters (Fig. 3), with minor physical deformation of the particle.

The nature of the impacts changed considerably for the 2.0 and 3.0 km s^−1^ velocity experiments. These highly destructive impacts had enough energy to initiate a phase change in the particles, causing substantial thermal disruption. Although residue from the particles was captured inside the craters, projectile mass is lost in the form of PMMA impact ejecta. This resulted in a decrease in capture efficiency across the CS materials, with the interesting exception of the 10 μm diameter particles, that suffered relatively poor capture efficiency for the lower velocity impacts. This suggests that thermal conductivity has little significance for capture efficiency during the highly destructive and energetic impacts, and that pliable materials capable of dissipating kinetic energy through target deformation are better suited to minimize ejecta and increase capture efficiency. This hypothesis is supported by the fact that gold and indium, the two softest CS materials with ultimate tensile strength of 120 and 4.5 MPa, respectively, had the highest capture efficiency for the 2.0 and 3.0 km s^−1^ velocity impacts.

Micro‐Raman spectroscopy was performed on the particles and residues captured during the impacts to determine whether the PMMA organic polymer suffered from significant bond disruption. Raman vibrational spectroscopy measures the symmetric vibrations of the polymer and significant bond disruption should alter the observed vibrational frequencies. A direct comparison between the Raman spectra of pre‐shot PMMA captured particles and residue revealed similar spectra. This result confirms that organic compounds remain chemically intact during impacts with velocity ≤2.0 km s^−1^. Raman microscopy was inconclusive for studying the 3.0 km s^−1^ velocity impacts due to the low capture efficiency; the residue was possibly too thin and had insufficient cross section to generate a detectable Raman spectrum. These are important results as they underline the significance of selecting an efficacious encounter velocity for organic sample capture during an Enceladus and potentially Europa fly‐by mission.

Impact speed alone is not the sole determinant of the outcome of an impact. The shock pressure generated in the impact is also important, and this varies at any given speed dependent on the properties of both the projectile and target. To gauge the peak shock pressure (PSP), we use the planar impact approximation (PIA; Melosh 1989). This approximation utilizes a linear shock wave relationship in the materials:

(1) U=C+Su

where *U* is the shock speed, *u* the particle speed, and *C* and *S* are constants. The values of *C* and *S* for PMMA, glass, and the target metals are listed in the supporting information. The PSP near the contact plane between the metal targets and PMMA and glass planes were calculated for 2 km s^−1^ velocity impacts. PMMA and glass were selected as ice analogs for the calculations to simulate ice that either melts or shatters on impact, respectively. Table 1 compares the calculated PSP of each target metal using the PIA for PMMA and glass. The results demonstrate that PSP is higher for glass versus PMMA, but there is only a modest variation between the different metals. The highest PSP were identified in gold, while the lowest were in aluminum for both PMMA and glass with an average difference of 4.5 GPa. The important result here is that there is not significant variation in PSP between the different target metals, and therefore, PSP is a negligible variable when selecting the optimal CS material for maximum organic survival. To complement this point, we compare the PSP from impacts between glass particles and similar metal plates at 2 km s^−1^ velocity using *AUTODYN* modeling data (interquartile maximum) (Golozar et al. 2018) and find the PSP between the different target metals correlates with results from the PIA calculations, despite having nominally lower values.

**Table 1:** Planar impact approximation (PIA) peak shock pressures (PSP) for planar PMMA and glass impacts compared with *AUTODYN* PSP for impacts between glass particles and metal plates at 2 km s^−1^ velocity. Specific target materials given in supporting information. AUTODYN is not parameterized for In impacts

Target material	PMMA PIA PSP (GPa)	Glass PIA PSP (GPa)	Glass AUTODYN PSP (GPa)
Ag	10.6	17.6	9.8
Al	8.5	13.3	6.5
Au	11.3	19.5	11.1
Cu	10.5	17.6	9.6
In	10.6	15.3	n.d.

## Conclusion

Capturing ice particles during Enceladus plume transits has been identified as a potential method of gathering pristine subsurface ocean samples from Enceladus for in situ chemical analysis. This work shows that capture systems in development can provide successful capture of intact organic ice‐particle simulants. These results also reveal how capture efficiency varies with particle size, impact velocity, and capture medium.

Any mission designed to collect samples from icy plumes must carefully consider the encounter velocity and capture medium of their collection instrument if high capture efficiency is desired. Our results indicate that optimal capture (~80% MRC) is achieved for particles with diameters ranging 4–6 μm and an impact velocity of ~1 km s^−1^ on indium foil. Under these conditions, the particles remain intact, both physically and chemically, and embed in the soft capture medium. Our demonstration that organic particles can be captured in high and hypervelocity impacts on certain CS without chemical modification is an important step forward.

A linear correlation was established between the impact velocity and crater diameter for the particles on the different CS materials. Data from these trend lines were then interpolated to provide particle–crater diameter calibration plots for each CS material at a given impact velocity. The particle–crater diameter calibration plots facilitate future ice particle impact experiments necessary for successful development of the EOA capture system. Impact experiments with ice particles entrained with organic compounds are currently being carried out and are providing important knowledge for the development of instruments capable of optimally probing for biosignatures in icy plumes at Enceladus and potentially Europa (New et al. 2019).

## Editorial Handling

Dr. Daniel Glavin

## Supporting information


**Table S1.** Supplier and catalog numbers for the CS metals used in the impact experiments.
**Table S2.** Mean residue coverage (MRC) data for the 4, 6, and 10 μm diameter particles on Ag, Al, Au, Cu, and In.
**Table S3.** Values for *y*‐intercept (a) and gradient (b) from the velocity–crater diameter plots in Fig. 8.
**Table S4.** Values of C and S for the CS materials, PMMA, and glass for different velocity ranges (Ahrens and Johnson 1995; Jordan et al. 2016).
