# Supplementary material for: Characterizing organic particle impacts on inert metal surfaces: Foundations for capturing organic molecules during hypervelocity transits of Enceladus plumes
Source: Meteorit Planet Sci. 2020 Feb 25;55(3):465–79. doi: 10.1111/maps.13448 (PMC7188319; doi:10.1111/maps.13448)
Supplement: Supplementary file 1 — Table S1. Supplier and catalog numbers for the CS metals used in the impact experiments. Table S2. Mean residue coverage (MRC) data for the 4, 6, and 10 μm diameter particles on Ag, Al, Au, Cu, and In. Table S3. Values for y‐intercept (a) and gradient (b) from the velocity–crater diameter plots in Fig. 8. Table S4. Values of C and S for the CS materials, PMMA, and glass for different velocity ranges (Ahrens and Johnson 1995; Jordan et al. 2016). [file MAPS-55-465-s001.docx]

Table. S1. Supplier and catalog numbers for the CS metals used in the impact experiments.

| Material | Supplier | Catalog # |
| --- | --- | --- |
| Ag | Goodfellow | AG000305/4 |
| Al | Goodfellow | AL000630/11 |
| Au | Goodfellow | AU000345/104 |
| Cu | Advent | CU130118 |
| In | Goodfellow | IN000260/15 |

Table S2. Mean residue coverage (MRC) data for the 4, 6 and 10 µm diameter particles on Ag, Al, Au, Cu and In.

| Particle diameter (µm) | Velocity (km s^-1^) | Ag (%) | Al (%) | Au (%) | Cu (%) | In (%) |
| --- | --- | --- | --- | --- | --- | --- |
| 4 | 0.547 | 66.0 | 21.0 | 53.0 | 31.0 | 46.0 |
| 4 | 0.851 | 85.0 | 49.0 | 84.0 | 46.0 | 82.0 |
| 4 | 2.01 | 20.4 | 16.6 | 4.6 | 3.7 | 16.9 |
| 4 | 2.68 | 5.4 | 5.4 | 2.0 | 3.2 | 7.5 |
| 6 | 0.425 | 4.0 | 4.0 | 4.0 | 4.0 | 4.0 |
| 6 | 0.995 | 9.0 | 3.0 | 57.0 | 6.0 | 79.0 |
| 6 | 1.95 | 12.8 | 7.5 | 14.5 | 14.9 | 15.0 |
| 6 | 2.93 | 0.2 | 0.1 | n.d. | 0.5 | 1.3 |
| 10 | 0.527 | n.d. | 2.3 | n.d. | 2.3 | 2.3 |
| 10 | 0.979 | n.d. | n.d. | 9.6 | n.d. | 18.5 |
| 10 | 1.94 | 30.8 | 14.7 | 27.7 | 19.1 | 35.3 |
| 10 | 2.92 | 0.3 | 0.04 | 0.2 | 0.1 | 2.2 |

Table S3. Values for y-intercept (a) and gradient (b) from the velocity-crater diameter plots in Fig. 8.

| Material | Particle diameter (µm) | a | b |
| --- | --- | --- | --- |
| Ag | 4 | 2.1143 | 1.4361 |
| Ag | 6 | 2.1426 | 2.5825 |
| Ag | 10 | 5.92 | 3.3978 |
| Al | 4 | 2.3899 | 1.8643 |
| Al | 6 | 3.3064 | 2.8903 |
| Al | 10 | 7.1776 | 4.0704 |
| Au | 4 | 3.1794 | 0.6789 |
| Au | 6 | 3.4981 | 1.4988 |
| Au | 10 | 6.6471 | 2.4762 |
| Cu | 4 | 2.7821 | 1.0564 |
| Cu | 6 | 2.9863 | 2.1176 |
| Cu | 10 | 4.8844 | 3.7073 |
| In | 4 | 4.1872 | 2.0282 |
| In | 6 | 6.5279 | 3.0496 |
| In | 10 | 7.9559 | 7.0615 |

Table S4. Values of C and S for the CS materials, PMMA and glass for different velocity ranges (Ahrens and Johnson 1995; Jordan et al. 2016).

| Material | C (km s^-1^) | S | U_p_ range (km s^-1^) |
| --- | --- | --- | --- |
| Ag | 3.23 | 1.59 | 0 – 2.149 |
| Ag | 3.23 | 1.59 | 2.12 – 4.32 |
| Al | 5.44 | 1.324 | 0.428 – 6.0 |
| Au | 2.95 | 1.81 | 0 – 0.71 |
| Au | 3.08 | 1.546 | 0.71 – 3.52 |
| Cu | 3.982 | 1.460 | 0 – 12.1 |
| In | 2.54 | 1.49 | 0.56 – 2.93 |
| In | 5.48 | 0.47 | 2.93 – 4.87 |
| PMMA | 2.766 | 1.365 | n.d. |
| Glass | 3.96 | 0.57 | 1.44 – 2.397 |
